# Integrating human rights into sexual and reproductive health research: moving beyond the rhetoric, what will it take to get us there?

**DOI:** 10.1080/26410397.2021.1881206

**Published:** 2021-02-17

**Authors:** Sofia Gruskin, William Jardell, Laura Ferguson, Kristin Zacharias, Rajat Khosla

**Affiliations:** a Director, USC Institute on Inequalities in Global Health, University of Southern California, Los Angeles, CA, USA. *Correspondence*: gruskin@med.usc.edu; b Program Specialist, USC Institute on Inequalities in Global Health, University of Southern California, Los Angeles, CA, USA; c Director, Program on Global Health and Human Rights, USC Institute on Inequalities in Global Health, University of Southern California, Los Angeles, CA, USA; d Research Associate, USC Institute on Inequalities in Global Health, University of Southern California, Los Angeles, CA, USA; e Human Rights Advisor, World Health Organization, Geneva, Switzerland

**Keywords:** sexual and reproductive health, human rights, rights, SRH, rights-based research, rights-based approach

## Abstract

The integration of human rights principles in sexual and reproductive health (SRH) research is often recognised to be of value. Good examples abound but lack of clarity persists as to what defines rights-inclusive SRH research. To help move the field forward, this article seeks to explore how key stakeholders responsible for funding and supporting rights in SRH research understand the strengths and weaknesses of what is being done and where, and begins to catalogue potential tools and actions for the future. Interviews with a range of key stakeholders including international civil servants, donors and researchers committed to and supportive of integrating rights into SRH research were conducted and analysed. Interviews confirmed important differences in what is understood to be SRH rights-oriented research and what it can accomplish. General barriers include lack of understanding about the importance of rights; lack of clarity as to the best approach to integration; fear of adding more work with little added benefit; as well as the lack of methodological guidance or published research methodologies that integrate rights. Suggestions include the development of a comprehensive checklist for each phase of research from developing a research statement through ultimately to publication; development of training modules and workshops; inclusion of rights in curricula; changes in journal requirements; and agreement among key funding sources to mandate the integration of rights principles in research proposals they receive. As a next step, cataloguing issues and concerns at local levels can help move the integration of human rights in SRH research from rhetoric to reality.

## Résumé

L’utilité de l’intégration des principes des droits de l’homme dans la recherche sur la santé sexuelle et reproductive (SSR) est reconnue. Les bons exemples abondent, mais un manque de clarté persiste sur la définition de la recherche sur la SSR inclusive des droits. Pour aider à progresser dans ce domaine, l’article se demande comment les principaux acteurs responsables du financement et du soutien des droits dans la recherche sur la SSR comprennent les forces et les faiblesses de ce qui est fait et où, et il commence à répertorier les outils potentiels et les mesures pour l’avenir. Des entretiens ont été réalisés et analysés avec un éventail d’acteurs clés, notamment des fonctionnaires publics, des donateurs et des chercheurs qui approuvent et soutiennent l’intégration des droits dans la recherche sur la SSR. Ils ont confirmé d’importantes différences dans ce qui est compris comme une recherche sur la SSR axée sur les droits et ce qu’elle peut accomplir. Les obstacles généraux comprennent le manque de compréhension de l’importance des droits ; l’insuffisante clarté quant à la meilleure approche de l’intégration ; la crainte de créer davantage de travail pour de faibles avantages ajoutés ; de même que le manque de conseils méthodologiques ou de méthodologies de recherche publiées qui intègrent les droits. Les suggestions comprennent la mise au point d’une liste de contrôle exhaustive pour chaque phase de recherche depuis l’élaboration de l’énoncé de la recherche jusqu’à la publication ; la préparation de modules de formation et d’ateliers ; l’inclusion des droits dans le programme d’études ; les changements dans les conditions des revues spécialisées ; et la volonté des principales sources de financement de rendre obligatoire l’intégration des principes des droits dans les propositions de recherche qu’elles reçoivent. En tant que prochaine étape, l’inventaire des problèmes et des préoccupations aux niveaux locaux peut aider à faire passer l’intégration des droits de l’homme dans la recherche sur la SSR de la théorie à la pratique.

## Resumen

La integración de los principios de derechos humanos en investigaciones sobre salud sexual y reproductiva (SSR) es reconocida como valiosa. Abundan los buenos ejemplos, pero persiste la falta de claridad en cuanto a qué define una investigación sobre SSR inclusiva de derechos. Con el fin de que progrese este campo, este artículo busca explorar cómo las partes interesadas clave responsables de financiar y apoyar los derechos en las investigaciones sobre SSR entienden las fortalezas y debilidades de qué se está haciendo y dónde, y empieza a catalogar posibles herramientas y acciones para el futuro. Se realizaron y analizaron entrevistas con una variedad de partes interesadas clave, tales como funcionarios, donantes e investigadores internacionales comprometidos a integrar los derechos en las investigaciones sobre SSR. Las entrevistas confirmaron importantes diferencias en lo que se entiende como investigación sobre SSR orientada hacia los derechos y qué se puede lograr. Entre las barreras generales figuran: la falta de comprensión sobre la importancia de los derechos; falta de claridad en cuanto al mejor enfoque para la integración; temor de agregar más trabajo con poco beneficio adicional; así como la falta de orientación metodológica o metodologías de investigación publicadas que integran los derechos. Algunas sugerencias son incluir la creación de una lista de verificación integral para cada fase de la investigación, desde la elaboración de la declaración de la investigación hasta la publicación; la creación de módulos y talleres de capacitación; la inclusión de derechos en currículos; cambios a los requisitos de revistas; y el acuerdo entre las principales fuentes de financiamiento de exigir la integración de los principios de derechos en las propuestas de investigaciones que reciben. Como un próximo paso, catalogar los problemas y las preocupaciones a nivel local podría ayudar a llevar la integración de los derechos humanos en las investigaciones sobre SSR de la retórica a la realidad.

## Background

It is often stated that integrating human rights in sexual and reproductive health (SRH) policies, programmes and services is essential to achieving the 2030 Agenda for Sustainable Development. Sustainable Development Goal (SDG) 3 “Ensure healthy lives and promote well-being for all at all ages” and SDG 5 “Achieve gender equality and empower all women and girls” both include targets that call for universal access to SRH services and realisation of relevant rights.^1^ The complex interplay of rights-related factors generally recognised to impact SRH outcomes includes the availability, accessibility, acceptability and quality of health services; informed decision making, privacy and confidentiality in the provision of health services; and nondiscrimination and equality with particular attention to key and marginalised populations.^2,3^ SRH-related outcomes are all further helped or hindered by the unique legal and policy environment and the larger economic, social, cultural and political determinants of local contexts, including the presence or absence of accessible and functional accountability mechanisms. Attention to all these components forms part of what is considered a rights-based approach to SRH.^4^

What this means for the research needed to put these policies and programmes into place is less clear. Previous writings have discussed how incorporating human rights concepts in SRH research can help

*“address power imbalances within and between institutions and programs, ensure transparent, inclusive and ethical research processes, enhance good governance of research institutions and promote research programs of particular relevance to people living in poverty/under oppression, women and marginalised groups while not compromising on quality”.*^5^

In practical terms, the extent to which the range of rights considerations noted above form part of SRH research is not well documented, even in research that claims to have a focus on sexual and reproductive health and rights. It is also not clear from a methodological perspective what such a commitment means for each phase of research, from the development of a research question, through to implementation, analysis and publication.^6^

A number of issues can be raised from the outset. How does one determine what qualifies as rights-based SRH research? Is it integration of rights at one or at every stage of the research process? Which rights are included? Is it the legal definition of rights or simply a concern with justice and equality? To qualify as rights-based SRH research, how relevant is the content or subject matter being addressed through the research? Is a focus, for example, on addressing inequalities among various population groups in their access to certain services necessarily rights-based?^5^ Must rights principles, such as participation or accountability, be explicitly adopted as such, forming part of the study question and, in turn, how each phase of the research is designed and implemented?^7,8^ Must the research team include lawyers or others with substantive expertise in rights? And how relevant is the final outcome under consideration – does it matter if the research is focused directly on, for example, increasing access to contraception versus a focus on improving the overall human rights situation for adolescents and young women, which over time will be assumed to improve access and use of contraception?^6^

There is no one size fits all, nor should there be, but a number of issues must be considered. Without setting out to explicitly answer each of the questions noted above, we set out to determine the potential factors that may help or hinder efforts to integrate rights into SRH research in practice, with a focus on understanding what is being done and how, and not only the successes, but the challenges faced in integrating rights into research. A literature review found that the integration of rights has yet to be comprehensively explored in the literature, even as confronting these issues and addressing them head-on seem to be recognised as of critical importance to moving the field forward. Identified barriers to implementing rights in SRH programming included broad structural, policy and health systems barriers as well as perceived financial cost, staffing and time constraints and a lack of understanding of how concretely to include human rights, while facilitators included the existence of human rights champions and leadership, strong civil society participation, training, guidelines and funding made available specifically for implementation.^9^ Additional key issues identified included the understanding of what human rights are and what they offer, awareness of appropriate methodologies, political will, the need for an enabling environment and clear accountability mechanisms.^9^ Identified barriers and best practices were utilised to set the parameters for this study.

This article explores the current status of rights integration in SRH research through key informant interviews to understand what is being done and where, successes and barriers to wider adoption of rights in SRH research, and to begin to consider tools, approaches and other actions that might be useful. These interviews are used as background for the analysis and discussion that follow.

## Methods

This section describes the methodology used to select key informants as well as the approach taken to data collection and analysis.

### Participant selection

Without aiming for saturation, 10 key informants centrally engaged in and committed to integrating rights into SRH research and/or relevant programming from a variety of disciplinary and organisational perspectives were interviewed between April and August 2019. These included donors, international civil servants (e.g. policymakers, programme implementers) and researchers, with both personal and organisational commitment to integrating rights within their work, including from the United Nations, national governments and international funding agencies.

### Data collection

Interviews were conducted by a trained interviewer using an interview guide developed for this purpose by the research team. The objective of these interviews was to determine how key stakeholders generally understand the quality and approach to what is being done and where, barriers they see to wider integration of rights in SRH research and tools and other actions they think would be useful to better incorporate rights into every stage of SRH research from the definition of the research question through to implementation, data analysis and publication. Detailed notes were taken during the interviews to capture content reported by informants.

### Data analysis

Capturing key points emergent in the interview data, notes from the 10 interviews were analysed looking for examples of current rights integration as well as challenges and successes in integrating rights at each phase of the research process. An iterative process of analysis was conducted. The literature review noted earlier was used to frame and analyse interview data, including attention to potential tools and approaches to take this work forward.^9^

## Results

Respondents provided general reflections on all that it takes to incorporate rights into SRH research including substantive and methodological challenges. They also noted overarching questions and approaches that they thought might be helpful to move the field forward. The findings presented below include salient quotes to illustrate the points being made.

In the first instance, several ways in which attention to rights can help to strengthen SRH research were noted by respondents. These included attention to power and power dynamics, understanding the condition and position of research subjects (whatever the topic), substantive attention to inequalities, and the potential to use the legal grounding of rights for subsequent advocacy and potential policy change resulting from research findings.

*“(Rights) strengthens (research) because it systematically draws attention to the things that we think matter that aren’t often explicitly called out in research and you can tell why things are or are not happening.”* (KII7, Researcher)

*“Power dynamics affect every part of a person’s experience including how the research is done and perceived, and I don’t think we’re as thoughtful about that as we need to be from the research perspective.”* (KII4, Funder)

Nonetheless, a concern raised by one key informant, and echoed by others, centred on the view of many in the SRH research community that rights are political, legal, and/or are not helpful to SRH research unless the research is expressly concerned with addressing a rights concern such as gender inequality.

*“The weaknesses are that human rights are in the first instance assumed to be political and people are scared of them, especially governments. Making clear to people that rights can be helpful to get to their outcomes is key to moving this work forward.”* (KII7, Researcher)

Respondents also described a general unwillingness among many SRH researchers who fear that the addition of rights to their research may be too expensive, too fuzzy, or take too long.

*“It’s considered a second-level priority unless the study is looking at that [rights] explicitly.”* (KII1, Senior International Civil Servant)

According to respondents, limited knowledge of sound methodological approaches for how to incorporate rights into research has also hindered their ability to take hold. For example, even as contraception, abortion and maternal health are areas where rights have been more or less successfully integrated from a programming perspective, this has not translated into research efforts, even within these same areas.

*“There was some work done around contraception, abortion, and maternal health but that was in programming and it’s not something that researchers would consider a natural part of their work.”* (KII3, Retired Senior International Civil Servant and Funder)

Furthermore, it was noted that some researchers think that, because they have a good heart and a general concern with social justice, they are already incorporating rights into SRH research, adding to further confusion about what is actually needed to integrate rights into SRH research effectively. When rights integration has happened systematically, respondents shared that it tends to be associated with specific donors.

*“The weakness is methodology, and awareness of the methodologies that do work. Integrating and unpacking on a granular level tends to be very nascent at this stage … When it does happen, it is largely focused on certain geographic areas funded by certain donors only … ”* (KII6, International Civil Servant)

The importance of both funders and ethical review boards was a recurring theme. Respondents discussed how the interests of donors or the strength and focus of ethical review boards play a role in how strongly human rights concerns or protections are dealt with when developing and implementing a research study. It seems that, even with the best of intentions, there are few funders or review boards who fully know how to engage rights in research despite general interest in doing so. Even when funders or review boards insist on attention to rights, there are researchers who lack a commitment to rights and simply pay lip service to rights in their initial conceptualisation because they are focused on getting the research through, and not necessarily because they see the added benefit of doing so. Respondents also noted that even when there is a commitment to rights at the time of the initial conceptualisation of a research project, they would often be forgotten as the work moved forward.

*“I have not seen many protocols that explicitly mention human rights and include them from the very start and all the way through the structure of the project. However, rights are very often mentioned in the background … It’s mentioned as something that is helpful and important, but it’s rarely incorporated into the design.”* (KII2, Senior International Civil Servant)

*“As a researcher, if you’re not asked for ‘the why’, then you’re not going to look for those answers.”* (KII9, International Civil Servant)

*“ … . sometimes when you don’t talk about something you forget about it and then that really biases how you’re understanding the problem. You forget that it’s a part of the equation … It’s like this with rights.”* (KII4, Funder)

Even when researchers have a commitment to bringing rights into the operational phases of their research, substantive barriers remain concerning content and actual implementation. One interviewee discussed the importance of having rights-focused indicators for data collection embedded in each phase of the study design to ensure attention to rights can be carried all the way through.

*“One of the challenges or barriers in the incorporation of each right per se is that we totally lack the measurements and don’t collect enough data on the context in which we are working or the people we are working with to make an assessment as to if, for example, we are discriminating or leaving people out.”* (KII4, Funder)

Another barrier noted to ensuring that rights-oriented methodologies that do exist become known and can be replicated concerns the limited appetite of many peer-reviewed journals to publish the details of the sorts of methodologies needed to incorporate rights into research, resulting in rights being less apparent in published work.

*“The bias is in the epidemiological framing. If we do an analysis that brings in rights considerations, getting this paper into X or Y journal may often be more in the editorial section than in the peer-reviewed, biomedical research section.”* (KII9, International Civil Servant)

Respondents generally made the point that, even with the best of intentions, the ability to integrate rights into research requires training and experience. They emphasised that any such training needs to make clear not only the need to draw attention to the reasons for paying attention to rights and the potential value added, but the concrete ways rights can be integrated into the different phases of research.

*“With gender and human rights as you go beyond what people have learned and it is less familiar this will require more time.”* (KII3, Retired Senior International Civil Servant and Funder)

*“It’s been a steep learning curve for me … I’ve learned so much in the past few years and I think people at the major public health places need to do the same. As I’ve come to understand a human rights perspective … I have learned a lot: what it is, what it means to apply it, etc.”* (KII1, Senior International Civil Servant)

Respondents provided a number of recommendations they saw as useful next steps to facilitate SRH researchers’ ability to bring rights into the different phases of SRH research, from approaches to developing research objectives to the sorts of tools and actions needed at every stage of the research process.

Interviewees discussed as a first order of business the need to establish a willingness among researchers to learn and develop the necessary skills and suggested messaging in a variety of fora on the added value rights could bring to their work.

*“There is a need for good public health research in the first place. And then what difference does it make using a rights-based approach to the study design or research questions? What are the underlying determinants, power structure? These questions need to be embedded in the study design. This starts to highlight the nature of tools that are required; training programs, facilitated online platforms that are specific to guiding people through all phases or research.”* (KII6, International Civil Servant)

Informed consent was discussed as a potential entry point for communicating with researchers the importance of rights integration, in that even if they are unfamiliar with how to integrate rights into their work, they are aware of the importance of informed consent processes.

*“One of the key things is informed consent. Regardless of if the research is biomedical or qualitative, this is the human rights aspect that most of the researchers try to cover also in terms of confidentiality, giving information, etc. Maybe this is a starting point where you can connect with all of them [the researchers]. Informed consent could be the common ground.”* (KII10, International Civil Servant)

One key informant, an international civil servant, suggested a methodology to help researchers conceptualise a three-tiered approach to rights in developing their research statement: including attention to contextual factors, the questions to be asked, and the population to be addressed, as a way to explicitly incorporate rights in ways that can impact all aspects of the research. It was noted that this would help researchers to think more broadly than research ethics by bringing attention to the larger contextual factors or environment where the research will take place.

Key informants suggested a variety of approaches as to how to take the integration of rights in research forward. Respondents discussed important considerations including tools, products and approaches including a checklist, trainings, and changes to university curricula and funding requirements by donors.

The development of a tool, such as a checklist with an accompanying training module, was discussed by several respondents as something that could be helpful and useful throughout each stage of research, even without previous human rights training. Respondents extrapolated what such a checklist might look like, and that it could, for example, include a process for establishing the rights most important to a particular SRH research question. Utilisation of a checklist throughout the phases of the research process was described as an easy step for researchers to follow for rights integration, so long as it was as specific as possible.

*“I think a checklist and module would be great. I think the complement to this would be to routinely have a discussion of these issues. If there are things included, perhaps in the checklist, you might suggest that a certain type of person be brought into the conversation. A checklist would flag issues to consider, the module would give expertise, and the discussions would supplement.”* (KII2, Senior International Civil Servant)

*“The more specific one can be in a checklist the better. How to address a specific human right. How to write it into a protocol and what is important. Take the most important human rights as relevant to a specific area of research and why they are important, and how to address each specifically in the protocol.”* (KII3, Retired Senior International Civil Servant and Funder)

Another interviewee, in responding more generally, noted that a checklist can help ensure that rights concerns become part of implementation throughout the research process. Another, in discussing the importance of ensuring rights principles are explicit in all phases of the research, noted that for this to be carried through successfully would require consistent check-ins between senior investigators with the research team on the ground to ensure these principles were not inadvertently ignored, and that there was the attention to the potential need for additional trainings as new issues came up (KII5, Researcher).

*“I would value some sort of checklist to know what I should be thinking about at each stage of research so that when I write proposals and implement my research, I have in mind what extra steps might need to be incorporated into the project design. At each stage of research it would be helpful to be reminded of rights and how to implement them.”* (KII7, Researcher)

It was noted that a short training may facilitate the incorporation of human rights principles or any tools such as a checklist. Respondents shared that laying the foundation for research questions, implementation, data analysis, findings and publication through a human rights lens could be a key component of a training. A training module was described by interviewees as being necessary to ensure that researchers are familiarised with what rights actually offer when attempting to include rights in each phase of research.

As an entry point, one interviewee pointed out that starting by offering training to those who are least resistant and most interested can be a first step in facilitating the integration of rights into research, as it will be necessary to convince researchers that this time is well spent.

*“Well-structured 1-2-hour online training courses can be done well and then are very useful if they are directly linkable and could be directly used in developing a protocol/research question. You can say to people this is how you should analyze your data to make sure that you’re including those principles. A checklist that is linked to an online training that is done because they have to, not just because they are interested – this may have a lasting impact.”* (KII1, Senior International Civil Servant)

*“People must see that it’s something of value to their research. Perhaps include a certification process and incentives as this will also help people who aren’t initially open get involved.”* (KII9, International Civil Servant)

Respondents also discussed the pros and cons of bringing in rights specialists to facilitate the efforts of research teams to engage in rights integration at the stage of proposal development, as well as once a proposal has been funded. While it was generally agreed that bringing in a specialist is not ideal, it was recognised to be an efficient way to help researchers become more aware of how to integrate rights principles into their work.

*“You want it to be a part of the broader thinking, but I think you would need a human rights specialist or someone who has been through a crash course [on rights].”* (KII1, Senior International Civil Servant)

Requiring a human rights specialist on in-country teams may not be possible, therefore other methods including the checklist and requisite training would ideally be stand-alone and not require a human rights expert as a full member of the research team. While none of those interviewed currently work at the local level, there was general agreement on the need to develop local expertise in human rights among public health researchers, and that such an exercise might expand the pool of people committed and able to bring rights effectively into SRH research efforts.

Another, longer term, aspect that respondents brought up was a need for universities and academic programmes training public health students to include human rights training, in particular as it concerns research and monitoring and evaluation in health, in their curricula. When students graduate, it was noted that their ability to understand the value of human rights principles to their research work would help to shape the work they would do once in a job.

Finally, respondents pointed out that if funding bodies require a rights perspective not only in proposals submitted but in the eventual write-up of research findings, researchers might be more inclined to learn more about how to incorporate rights into their work. As funders were described as having an obligation to ensure things are done per established and funded research protocols, an important first step would be to educate funding bodies on the importance of rights in the implementation of health research, and not simply as part of the background statement. They, in turn, would be able to educate researchers on doing proper rights-based work, as well as provide ongoing support, including review committees to ensure implementation is done as prescribed.

*“Working with funding bodies … to structure a capacity-building training online that would help the applicant that would include why it’s important in addition to how to do it. It’s the low hanging fruit, people need that information. Maybe going to the key donors and giving them an orientation, get them up to speed as well. As researchers, that would create a motivation because they have to have a protocol the funder will approve.”* (KII1, Senior International Civil Servant)

*“It’s hard to check that researchers are actually doing what is in the protocol. Having some committees review the protocol would be helpful.”* (KII3, Retired Senior International Civil Servant and Funder)

## Discussion

The interviews confirm that even as the need for the integration of rights in SRH research is fairly well established, there is still a general lack of clarity about what this means in practice. SRH research that takes rights into account spans broad social justice framing, very legalistic approaches to rights, and everything in between. This makes it clear that tools and training are needed to support the integration of rights in SRH research that do not dictate only one way of doing things but offer processes that are sufficiently malleable to support the range of approaches that do exist, and, importantly, with respect to every step of the research process. As it appears many in the larger SRH research community still do not see the value of rights for their work, there is a need also for attention to social media and other communication strategies that can highlight the added value of attention to rights for research and outcomes, as well as outreach to journal editors to support publication of relevant research methodologies. Further, it is clear that to truly support rights in SRH research will require larger structural changes, from what is taught in schools of public health to the approaches taken by funding agencies and other large institutions in what they do concretely to support the integration of rights.

Many of the issues that surfaced in previous literature reviews were found to translate to SRH research, but additional issues surfaced including a lack of understanding of how to operationalise specific human rights principles, and how to use rights not only as part of the conceptual background to research being undertaken but to practically support research questions and implementation of a research study, as well as the approach taken to data analysis and writing up the work for publication.^9^ Further points noted in the literature, and reiterated through the interviews, include the short timeframes imposed by many donors for demonstrating research outcomes, that inhibit the ability to truly understand and write-up the longer-term changes that might be possible through the inclusion of rights.^6^

The recommendations provided by key informant interviews can provide an important starting point for the content of any tools, guidelines or training materials to be developed. Tangibly, a key need identified through the interviews was a checklist, with appropriate training support, that could provide hints or questions for how to integrate rights at each phase of research without attempting to enforce a one-size-fits-all approach. Substantively it was nonetheless clear that certain issues would be relevant to all SRH research, such as attention to power dynamics, including within the research team, and questions of how and why rights are being integrated at each phase, as well as the potential to use research findings based on a rights framework for advocacy and policy change further down the line.

Each of the recommendations that emerged from these interviews would require the development of a robust and carefully thought through and tested strategy so as not to overwhelm researchers, funders or others seeking to better integrate rights into research. The implementation of any tool, such as a training or checklist, would also require careful monitoring to ensure they positively impacted the research process, did not unnecessarily impact cost, workload or personnel, and contributed beneficially to intended outcomes.

For years, there have been those who juxtapose a public health approach against a human rights approach as though the two are distinct and seek to do different things. While patently false this perception nonetheless has particularly stymied integration of rights into research. Hence there is a need for appropriate documentation and effective communication strategies that can highlight research which demonstrates the added value of rights for achieving public health goals, thereby pushing the conversation past a focus on the theoretical advantages of rights integration to actually demonstrating to those who are sceptical what this means in practice.

To bring rights more concretely into SRH research will require both a top-down and a bottom-up approach working in tandem. Ultimately, funding agencies have a key role in shaping where, how, for how long, and even if, research projects that bring in rights can occur. Engaging with publishers, funders and larger institutions on concrete actions that will facilitate the ability of researchers to integrate rights into SRH research will be key, while simultaneously providing very specific guidance to researchers as to how to think about rights at every stage of the research process. Researchers are not likely to incorporate rights into their work in systematic and replicable ways when funding bodies and other agencies are not providing the requisite support for their ability to do so. An enabling environment, even if support for this sort of research exists among funding bodies and global institutions, is necessary for local level action that integrates rights. As a next step, additional analysis to catalogue issues and concerns at grassroots and local level would help ensure the development of tools for rights integration that would adequately address the range of challenges that exist.

## Limitations

The key informant interviews conducted provide a range of perspectives, even as they do not provide a comprehensive understanding of all that is happening in terms of rights-based SRH research. While ensuring the perspectives of high-level funders, civil servants and researchers have been captured, in order to capture a more robust understanding as to what is happening in the field and what is needed, additional interviews, particularly within local settings, could provide additional important insights.

## Conclusions

Attention to rights at each stage of SRH research must be deliberate. While this study focused on the perspectives of high-level funders, civil servants and researchers, additional attention to local level perspectives would be a key next step for integration of rights in research to be meaningfully taken forward. Utilising the research cycle as the entry point can move this conversation from more general commitments to specific methodologies, providing a level of granularity and methodological approaches for rights consideration from start to finish. Steps can be taken within each phase of research, from developing a research statement through ultimately, to publication, but this requires training and support of researchers seeking to do this work by global and national institutions, as well as organisational change in the curricula used within institutions of higher learning, the issues considered by institutional review boards, and the seriousness of the approach to rights taken by funding agencies and other donors.

Global guidance exists to help support the integration of human rights into the provision of SRH policies, programmes and care. However, little guidance exists that prioritises and supports the integration of human rights into the SRH research process, and few currently have the mandate or tools to support this work in replicable ways. A cataloguing of issues and concerns at the local level culminating in comprehensive global guidance, mandates, tools and trainings will help move the integration of human rights in SRH research from rhetoric to reality.
